# Metazachlor traces in the main drinking water reservoir in Luxembourg: a scientific and political discussion

**DOI:** 10.1186/s12302-017-0123-z

**Published:** 2017-09-15

**Authors:** Pol Karier, Georges Kraus, Isabelle Kolber

**Affiliations:** SEBES (Syndicat des Eaux du Barrage d’Esch-sur-Sûre), rue Lultzhausen, L-9650 Esch-sur-Sûre, Luxembourg

**Keywords:** Metazachlor, Drinking water, Artificial lake, European directive, Relevance criteria, Macrophyte, Cyanobacteria, Protection zone

## Abstract

This discussion is centralized around an incident that took place in the Belgian village Witry the 17th of September 2014. A tractor accident led to the discharge of an aqueous solution of the herbicide metazachlor into the creek *Moyémont* that further merges into the river *Sûre*. About 20 km downstream, these waters supply the lake of the Upper-Sûre in Luxembourg, the biggest artificial lake and the main drinking water reservoir in the country. The evolution of the concentration of metazachlor and its metabolite 479M08 was partially tracked down from the river *Sûre* to the dam situated in the east. At this location, the SEBES drinking water treatment plant has its raw water intake from the lake. After this incident, substantial pollution by the metazachlor breakdown product 479M08 of the lake and of some other groundwater sources in the Grand Duchy was revealed due to a strong monitoring program that was started by the national water authority (AGE). This was for example the case in the SEBES groundwater resource *Scheidhof close to Luxembourg City*. There is also the reason to assume that contamination by 479M08 existed already in the lake before the incident in Witry, certainly due to agricultural activity. In the second part of this discussion, these perceptions are placed in their appropriate political context. Indeed, the quality of groundwater and drinking water is strongly regulated in the European Union and in Luxembourg. Compound 479M08, for instance, is submitted to a maximum parametric value of 0.1 µg/L in Luxembourg. Several short- and longtime political measures had to be taken to guarantee the wholesomeness of the water from a legal point of view.

## Main text

### Background

**Fig. 1:**
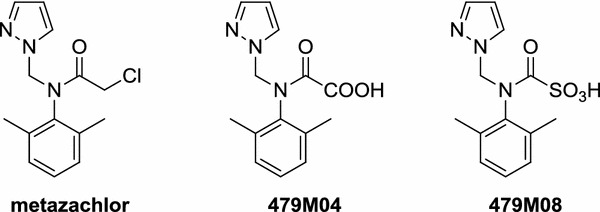
Structure of metazachlor and two of its metabolites

The *α*-chloroacetamide herbicide metazachlor (or 2-chloro-*N*-(pyrazol-1-ylmethyl)acet-2′,6′-xylidide in IUPAC nomenclature) is a cell division inhibitor that is widely used in agriculture for the pre-emergence control of broad-leaved weeds and annual grasses [1] (Fig. **1**). Metazachlor is mainly applied in winter and spring to *Brassicaceae* like rape, Brussels sprouts, cauliflowers, cabbage, and broccoli. At low concentrations in plants and algae, the herbicide acts as an inhibitor of the synthesis of very-long-chain fatty acids (VLCFAs), embodying more than 18 carbon atoms [2, 3]. A plasma membrane poorly supplied with these VLCFAs loses its rigidity and permeability, resulting eventually in the leakage of cell content and improper cell division [4]. Several other modes of action were reported and next to these primary responses, it is likely that higher concentrations of the *α*-chloroacetamide lead to additional secondary physiological reactions [5–9]. Although these herbicides have been in practical use for over 40 years [10], resistance problems of weeds against *α*-chloroacetamides are rare events [11]. This explains, at least partially, why this plant protecting agent is still so popular nowadays.

**Fig. 2:**
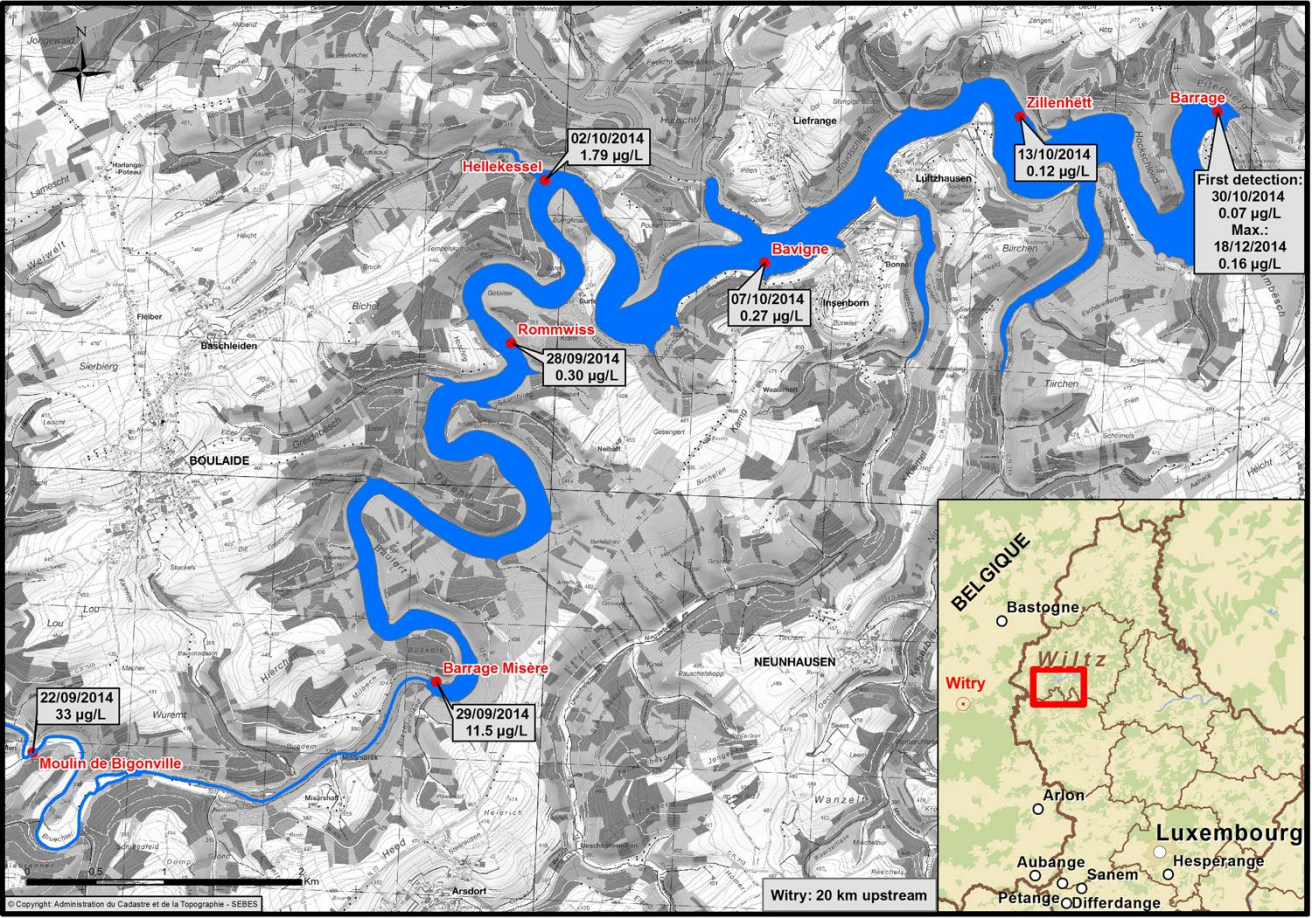
Situation of the lake of the Upper-Sûre and the measure points. Unless differently stated, the indicated values are the highest detected concentrations of metazachlor

The 17th of September 2014, a road accident of a tractor equipped with a boom sprayer led to the discharge of a ready-to-use aqueous solution of metazachlor (between 7.5 and 12.5 kg) and quinmerac (in 6000 L) onto the street in Witry (Belgium). In order to handle the chemical, the local fire workers rinsed the road with additional water (15,000 L), leading to significant run-offs into the creek *Moyémont*, further merging into the river *Sûre*. About 20 km downstream, the latter supplies directly the artificial lake of the Upper-Sûre in Luxembourg (Fig. 2).

Possessing a surface of 3.8 km^2^ (0.15% of the surface of country), an average depth of 16 m, and a total capacity of 60 × 10^6^ m^3^, the lake of the Upper-Sûre is the largest aquatic body in the country. In conjunction with being the main drinking water reservoir of the Grand Duchy, the lake is used for electricity production, flood protection, and environmental aspects. Furthermore, it is a preferred location for tourism and recreational activities. The local drinking water treatment plant SEBES (*fr.* Syndicat des Eaux du Barrage d’Esch-sur Sûre) that is installed next to the dam is able to supply 89% of the Luxembourgish population with drinking water, i.e., 20 × 10^6^ m^3^ drinking water per year [12]. The crude water can be extracted from the lake just ahead the dam at different depths, as a function of algal biomass distribution and manganese concentration in the water column. The water treatment plant is equipped with a multi-step purification system counting (in order of appearance) a front-end filter, an ozonation system, active coal treatment, Al^3+^ flocculation ponds, sand- and calcium carbonate filters, and a chlorinator. The drinking water peak-demand is not covered by the lake but is guaranteed by groundwater sources located at strategic points in the distribution network of the SEBES all across the country. Contrary to the water stemming from the lake of the Upper-Sûre, these additional sources are usually used without (major) treatment.

The aim of this current discussion is to report on a case from the field. We hope that this information can lead in similar situations to a better understanding, an improved prediction and a more accurate risk assessment of the pollution. This discussion also tends to bridge between environmental problems and European or national legislations. This particular case is an excellent illustration of the tight interconnection between politics and science, and highlights how fast this narrow equilibrium can be disordered.

### Discussion of the interaction of metazachlor with different compartments

#### Distribution of metazachlor in the lake of the Upper-Sûre

The evolution of metazachlor in the Upper-Sûre ecosystem was tracked by the SEBES, the AGE (*fr.* Administration de la gestion de l’eau), and BWL (*de.* Bergisches Wasser- und Umweltlabor) by HPLC MSMS (according to the German standard method DIN 38407-35) and is revealed in Fig. 2. The chloroacetamide is first detected in *Moulin de Bigonville* on the 22nd of September 2014, 5 days after the discharge in Belgium. At this place, the highest concentration of 33 µg/L was measured. In the next 2 weeks, the herbicide was transported with estimated average flow velocities of 1.5 km/day, steadily to *Barrage* over *Barrage Misère* (first detection: 23.09.14, 1.1 µg/L; highest detection: 29.09.14, 11.5 µg/L), *Rommwiss* (first detection = highest detection: 28.09.14, 0.30 µg/L), *Hellekessel* (first detection: 28.09.14, 0.23 µg/L; highest detection: 02.10.14, 1.79 µg/L), *Bavigne* (first detection: 05.10.14, 0.15 µg/L; highest detection: 07.10.14, 0.27 µg/L), *Zillenhëtt* (first and highest detection: 13.10.14, 0.12 µg/L) to *Barrage* (first detection: 30.10.14, 0.07 µg/L; highest detection: 18.12.14, 0.16 µg/L). In the rear of *Bavigne*, no more significant metazachlor concentrations could be identified (Fig. 2). This is due to several reasons including, most likely, (a) the high dilution factor and (b) dissipation and degradation mechanisms of metazachlor.

- Due to the morphology of the lake, the dilution at *Moulin de Bigonville* was still relatively poor. However, if we assume a complete and a homogeneous dissolution of the 7.5–12.5 kg of metazachlor in the entire lake (60 × 10^6^ m^3^), a concentration of 0.13–0.21 µg/L results.
- Further decrease in concentration derives from dissipation mechanisms. Although metazachlor is stable to aqueous hydrolysis and photolysis in environmentally relevant conditions (sunlight, pH, and temperature) [13], Mohr et al. showed that metazachlor concentrations decrease in the water column following a first-order curve with DT_50_ (median dissipation) values ranging from 37.4 to 47.9 days in lentic ecosystems, and from 27 to 44.2 days in lotic ecosystems [14]. In aerobic water–sediment studies, metazachlor disappeared with first-order DT_50_ values of 13.4 to 27.8 days for the whole system [13]. In these investigations, metazachlor dissipated from the water phase with DT_50_ values of 48.8–384 days, and from the sediment phase with DT_50_ values of 3.0–6.8 days.
  Metazachlor is only adsorbing moderately to soil, and the adsorption of the metabolites 479M04 and 479M08 is even weaker [13]. Consequently, the transportation of these substances from the water to the soil seems to be low. Metazachlor was degraded in the soil under field conditions with a mean DT_50_ of 6.8 days [13]. The two metabolites 479M04 and 479M08 were found in aerobic degradation studies of [^14^C-phenyl]metazachlor in soil at concentrations of 16 and 21% of applied radioactivity. These two metabolites were measured after 91 days and further dissipated with DT_50_ values of 56.4 and 71.1 days, respectively. Two further, minor metabolites were also detected (479M09 and 479M11).
  The dissipation of metazachlor and its metabolites from the aqueous phase to the air was assessed to be low [13].

**Fig. 3:**
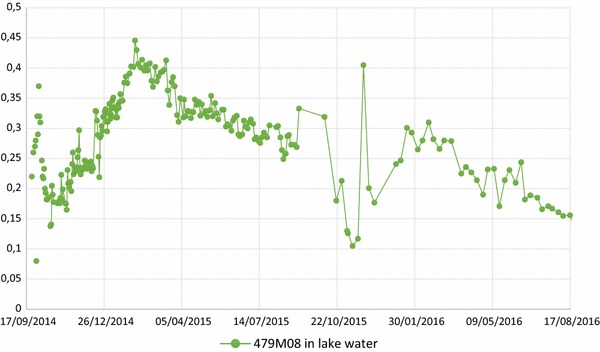
Evolution of the concentration of metabolite 479M08

Next to metazachlor, the fate of the metazachlor metabolite 479M08 was investigated in the lake of the Upper-Sûre. Concentrations of this breakdown product, ranging from 0.2 to 0.4 µg/L were found close to the dam, at every depth, mainly between the 3rd and the 7th of October 2014 (Fig. 3). Thereafter, the concentrations of this substrate dropped again to 0.15 µg/L. Quite surprisingly and completely unexpectedly, the concentrations then increased again to values between 0.3 and 0.45 µg/L during winter months in 2014 and early spring in 2015. It is unclear whether these raised concentrations were due to the accident in Witry, or resulted from continuous run-offs from agricultural activity that is particularly intensive for metazachlor during winter. Indeed, about 60 ha of the southern watershed of the lake are dedicated to the production of oilseed rape. A total surface of 100 ha in the Luxembourgish watershed is estimated to be devoted to this crop. Because the substance is mainly applied during cold months, it is very likely that the biodegradation is particularly slow, which enhances its potential for accumulation. The important mean slope around the lake, correlated with rainfall, further favors accretion in the water. Metazachlor (and its metabolites) are known to easily enter the aquatic environment by run-offs and these compounds have already been found in concentrations between 0.1 and 100 µg/L in European groundwater and surface water [15–18]. It is extremely likely that the contamination existed already in the lake of the Upper-Sûre before tracking these compounds and independently of the incident in Witry. The concentrations of 479M08 globally dropped during 2015. This is, most likely, a consequence of the legal decisions to prohibit the use of metazachlor that were taken (vide infra). The metabolite 479M04 was also found along with 479M08, but in much lower concentrations.

#### Risk for the drinking water consumers

Liver was shown to be the target organ in rats, mice, and dogs [13]. For mammalian, metazachlor acute toxicity is low via oral, dermal, and inhalation routes [LD_50_ >2000 mg/kg (median lethal dose) and LC_50_ >34.5 mg/L (median lethal concentration)]. Metazachlor did not show any genotoxic potential in a number of in vivo and in vitro studies. Metazachlor did not show any potential for acute, repeated dose, or delayed neurotoxicity, but the compound was considered to be carcinogenic at high dose levels (proposed classification carcinogenic category 3, R40 “Limited evidence of a carcinogenic effect”). An ADI (acceptable daily intake) of 0.08 mg/kg bw/day was derived from a NOAEL (no observed adverse effect level) of 8.5 mg/kg bw/day in the chronic rat study. The applied safety factor was 100. An ARfD (acute reference dose) of 0.5 mg/kg bw was derived from the NOAEL of 50 mg/kg bw/day, applying also a safety factor of 100.

#### Ecotoxicology

Metazachlor is classified as H400 and H410 designating acute and chronic aquatic hazard. Algae and higher aquatic plants, possessing high and constant amounts of VLCFAs are the most prone organisms to damage. Mohr et al, for example, showed that single exposure of several aquatic macrophytes to metazachlor at nominal concentrations of >5 µg/L, had pronounced long-term effects on the aquatic biota [14]. In this study, even after 140–170 days, the macrophytes did not recover from punctual metazachlor treatments. Applied metazachlor concentrations of 20 µg/L in pond- and stream mesocosms led to a decrease in macrophyte wet weight of 25 and 77%, respectively. In the same study [14], it was shown that filamentous green algae dominated by *Cladophora glomerata* and *Spirogyra* spec. were also extremely sensitive to metazachlor with EC_50_ values of 3 and 9 µg/L (median effective concentration) in stream- and pond indoor mesocosms, respectively. These algae were replaced by the filamentous yellow–green algae *Vaucheria* spec, but only at metazachlor concentrations higher than the ones observed in the lake of the Upper-Sûre (>80 µg/L). It is also known that the presence of metazachlor has repercussions on physicochemical parameters like pH and oxygen saturation via mechanisms implying biomass degradation and photosynthesis [14]. Next to factors like competition, there is reason to assume that these variations in physicochemical water parameters are also on the origin of species shifts, due to the different requirements of the communities.

The Lake of the Upper-Sûre is regularly victim of the massive proliferation of harmful algal blooms being composed, to a big extend, of cyanobacteria (*Anabaena*, *Microcystis*, *Woronichinia*…). Already in 1974, routine controls in the laboratory of the SEBES revealed a shift towards a greenish water coloration combined to a strong deoxygenation in late summer. These were the first signs of eutrophication and in 1986, the water production had to be stopped due to massive proliferation of planktonic algae. During a study led by Wille, it was shown that the lake of the Upper-Sûre undergoes, in general, an annual cycle composed of four different phases that are each defined by the dominance of one main algal group (In order of appearance): cryptophytes, diatoms, chlorophytes, and cyanobacteria [19].

Although the successions of different algal populations are strongly dependent on nutrients (phosphor, nitrogen,…), mechanical constraints (flow velocity, convection,…), and biological factors (allelopathy,…), it is possible that there is, at least to some extent, an influence of metazachlor on population distributions in the lake of the Upper-Sûre due to changes in competition. This hypothesis is further corroborated by a study led by Mohr et al. who showed that chlorophytes were the most prone organisms to be harmed by metazachlor [20]. On the other hand, diatoms and cryptophytes seemed insensitive. It is possible that the selective action of metazachlor on the chlorophytes, preceding the cyanobacteria in time, provides an advantage to the proliferation of the cyanobacteria in the upstream dam and the first part of the lake of the Upper-Sûre. A similar shift from a dinoflagellate-dominated phytoplankton, to a community overshadowed by the blue algae *Anabaena flos*-*aquae* was monitored by Noack [21].

Although this sounds like a plausible rationale, care has to be taken with this assumption because algal distribution depends on a plethora of different parameters. Toxicological studies on aquatic organisms of metabolites 479M04 and 479M08 show that they are, at least three orders of magnitude, less toxic than the parent metazachlor. In general, the risk of these compounds is assessed to be of little importance [13]. Due to the two-dimensional nature of the topic (environment and legislation), we will now try to place the incident in Witry in its legal context.

### Political aspects

#### European directives and Luxembourgish legislations

In the European directive, regulating the quality of water intended for human consumption (EU Drinking Water Directive), it is stated that pesticides are organic insecticides, herbicides, fungicides, nematocides, acaricides, algicides, rodenticides, slimicides, related products (*inter alia*, growth regulators) and their relevant metabolites, degradation and reaction products [22]. Limit values for active substances in pesticides, including their relevant metabolites, degradation and reaction products are fixed at 0.1 µg/L for single substances, and 0.5 µg/L for the sum of substances.

Due to the tight interconnection of drinking water and groundwater, the latter is submitted to the same limit values (0.1 µg/L for single substances and 0.5 µg/L for the sum of substances, Groundwater Directive 2006/118/EC) [23]. Although these directives fix the limit values, no criteria for the definition of relevant and non-relevant metabolites were provided at that stage, leading to uncertainty for regulators and notifiers. To avoid further misinterpretations, a definition of the relevance criteria was finally delivered in 2000. According to the EU DG Sanco Guidance, a metabolite is relevant if:

- It has comparable biological target activity (≥50%) to the active substance, or
- It has toxicological properties that are regarded as severe or unacceptable (e.g., genotoxic, or classified as toxic or very toxic) [24].

Metabolites are defined as non-relevant if:

- they exhibit “clearly less biological activity” than the parent substance (<50%) and
- they are not genotoxic and
- they do not belong to the classification toxic/very toxic, R60, R61, R62, or R63 and
- they are not metabolites of compounds, which themselves are classified as R45, and where evidence has been provided that an R40 for a parent substance does not lead to a risk of carcinogenicity for the metabolite.

The definitions and assessments of relevance provided by the DG Sanco guidance are not legally binding for the EU member states.

The Luxembourgish transposition of the EU drinking water and groundwater directive into national law contains a direct translation of the EU text on pesticides and their relevant and non-relevant metabolites. The highest concentration of metazachlor allowed in the groundwater and drinking water is therefore 0.1 µg/L and the sum of the concentrations of all pesticides must not exceed 0.5 µg/L. Despite the low toxicity of the two major metazachlor metabolites 479M04 and 479M08, they are both currently considered relevant. This denomination is based on the proposed designation of the parent metazachlor as carcinogenic category 3 R40 (vide supra, point d). Henceforward, they are submitted to the same threshold of 0.1 µg/L. Because the treatment of the water at the SEBES plant allows to eliminate, on average, 90% of metazachlor and its metabolites (maximum concentrations of 0.45 µg/L at the dam), the drinking water after treatment was, by law, still suitable for distribution following the metazachlor incident in Witry.

#### Political consequences

However, for precautionary reasons, the SEBES plant stopped providing drinking water from the 3rd till the 7th of October 2014 and switched to the 15 alternative SEBES groundwater wells, providing 38,000 m^3^/day. This decision was based on the assumption that potential high peak doses of the pollutants could contaminate the plant. The water outflow at the dam was also increased to have a faster elimination of the metazachlor metabolites from the lake.

**Fig. 4:**
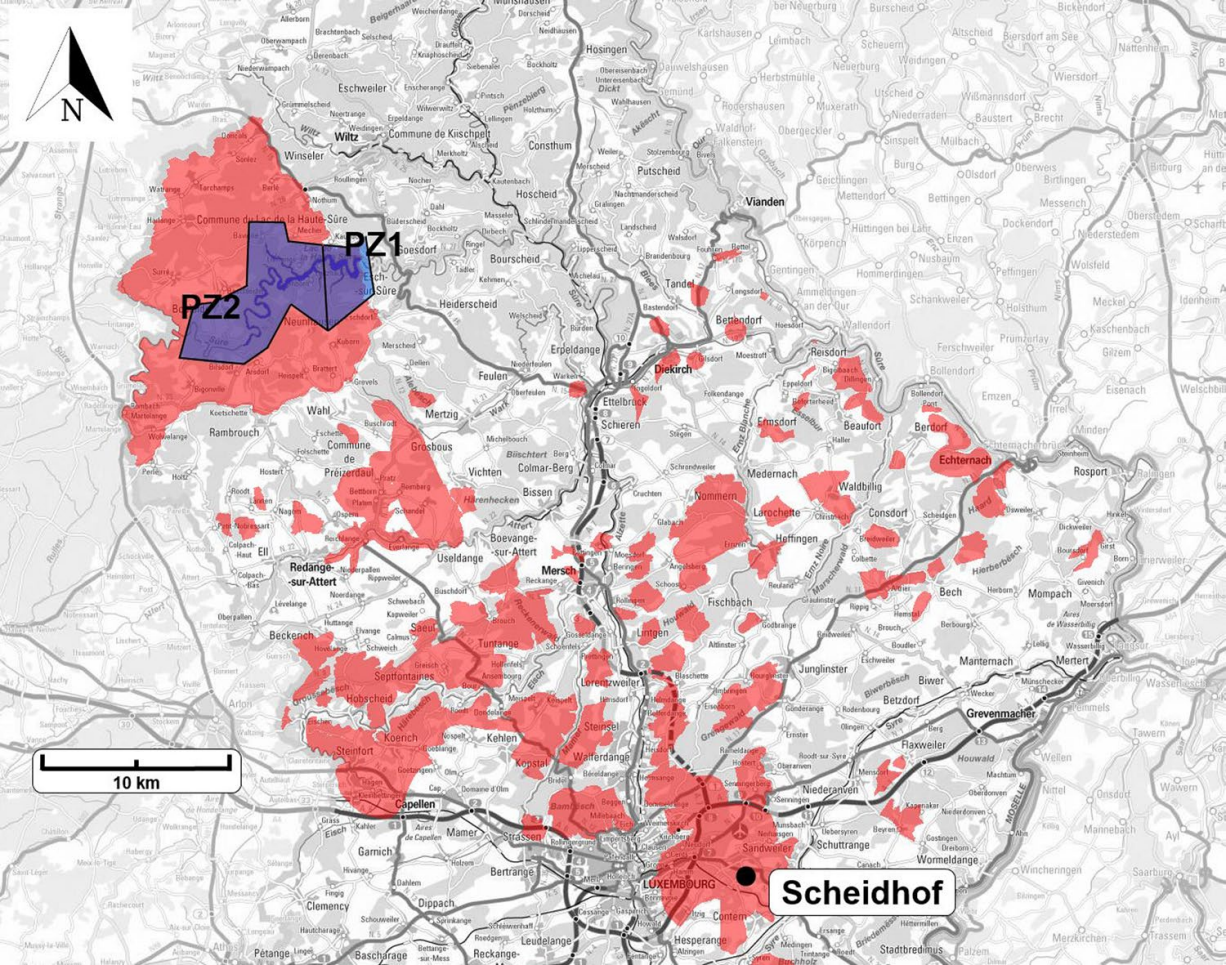
In *blue*: representation of the Protection zones 1 and 2 (PZ1 and PZ2) and in *red*: the areas where the use of metazachlor is prohibited

During this operation, it was noted that one of the main groundwater drilling wells: *Scheidhof* was also contaminated by the metazachlor metabolite 479M08 in concentrations up to 2–3 times higher than the allowed value of 0.1 µg/L (Fig. 4). A transition solution is now provided by article 11(4) of the Luxembourgish legislation on the quality of waters destined for human consumption [25]. This article claims that the supplier can ask for a 30-day exemption to distribute the water with non-respect of the parametric value (concentration of 479M08). However, the supplier has to guarantee that the non-respect of the threshold is of no consequence for human health. Furthermore, it has to be assured that the distributed drinking water meets the legally binding requirements again after 30 days. *Scheidhof* is kept as a back-up solution in case of water deficit. For some other contaminated groundwater sources, a dispensation is granted by article 11(3) in reciprocity of an extensive plan of action to restore the water quality within maximum 3 years.

In April 2015, the use of metazachlor became immediately and definitely banned in large surfaces of Luxembourg, including the protection zones 1, 2, the areas that are to become protection zones in the future (vide infra) and the entire supplying Luxembourgish watershed [26] (Fig. 4). Outside these zones, the use was regulated to 0.75 kg/ha, only 1 out of 4 years on the same surface. Prohibition to apply metazachlor in 2015 on surfaces on which it has been applied between 2012 and 2015 was also declared.

In order to face continuous pollution by means of run-offs, a new protection zone concept is currently being elaborated for the Upper-Sûre lake. To date, the protection zones that were defined by the national law of the 27th of May 1961 are still in use [27] (Fig. 4). In the new scenario, these two existing protection zones will be extended by a third zone. The protection areas 2 and 3 not only take into account the surface of the watershed (42,600 ha, amongst which 2/3 is situated on Belgian ground), but also consider the slope of the terrain. These protection zones will, in a first step, only be installed on Luxembourgish territories. Although this new concept, once concluded, should be a solution against continuous pollution from agricultural activity, accidents like in Witry are less likely to be prevented. The decline of these incidents is more likely to be promoted by different road-security regulations in critical areas. A cooperation (LAKU) intended to join the interests of the farmers and of the SEBES, was also founded in 2015 and mainly co-financed by the Luxembourgish Water Fund.

Besides the protection zones encompassing the lake of the Upper-Sûre, around 80 other protection zones that shall guarantee the longtime drinkability of the water from the different groundwater sources including *Scheidhof,* are currently under investigation. To a few exceptions close, these protection zones are almost entirely superimposable with the zones where the use of metazachlor is prohibited (Fig. 4). Some of these sectors were already defined in Luxembourgish legislations in January 2015 [28].

### Conclusions

The incident in Witry led to local concentrations of metazachlor and metazachlor metabolites in the lake of the Upper-Sûre that had the potential to harm, or at least, to alter the aquatic biota. The extent of these interactions is, however, due to limited amount of experimental data and the complexity of the ecosystem, not known. The risk for human water consumers was low due to the high dilution and the aptitude of the SEBES plant to eliminate between 70 and 98% of metazachlor and his metabolites content. This led to concentrations of the regulated substances in the drinking water (after treatment) that were below the legal European and Luxembourgish threshold of 0.1 µg/L. More disturbing is the observation that the groundwater reservoir *Scheidhof* was already contaminated by the relevant metabolite 479M08. Based on the moderate degradation of the parent metazachlor in pure aquatic media, it is possible that the breakdown of 479M08 in the groundwater wells is also extremely time consuming. Furthermore, corroborated by the evolution of the concentration of 479M08 after the Witry incident (Fig. 3), there is a high chance that the lake was already transporting similar high concentrations of 479M08, due to agricultural activity. Although the concentrations are still far away from toxicological thresholds for humans, it is important to point out and to remind the precautionary principle extracted from the Water Framework Directive that groundwater must be regarded as a natural resource, which should be protected in its own right [29]. Limit values for active substances and their metabolites should not be based exclusively on human toxicological values but should aim at a maximum preservation of the water. It is considered appropriate that an adequate level of protection is established for groundwater.

## Abbreviations

ADI: acceptable daily intake
ARfD: acute reference dose
bw: body weight
DT_50_: median dissipation time
EC_50_: median effective concentration
LC_50_: median lethal concentration
LD_50_: median lethal dose
NOAEL: no observed adverse effect level
SEBES: *fr.* Syndicat des Eaux du Barrage d’Esch-sur-Sûre
VLCFA: very-long-chain fatty acids
